# Inherited Disorders of Thyroid Hormone Metabolism Defect Caused by the Dysregulation of Selenoprotein Expression

**DOI:** 10.3389/fendo.2021.803024

**Published:** 2022-01-18

**Authors:** Kyu Won Lee, Yoochan Shin, Sungahn Lee, Sihoon Lee

**Affiliations:** ^1^ Department of Food Science and Engineering, Ewha Womans University, Seoul, South Korea; ^2^ Laboratory of Genomics and Translational Medicine, Department of Internal Medicine, Gachon University College of Medicine, Incheon, South Korea

**Keywords:** thyroid, thyroid hormone, selenium, selenoprotein, deiodinase, genetics

## Abstract

Consistent activation and functioning of thyroid hormones are essential to the human body as a whole, especially in controlling the metabolic rate of all organs and systems. Impaired sensitivity to thyroid hormones describes any process that interferes with the effectiveness of thyroid hormones. The genetic origin of inherited thyroid hormone defects and the investigation of genetic defects upon the processing of thyroid hormones are of utmost importance. Impaired sensitivity to thyroid hormone can be categorized into three conditions: thyroid hormone cell membrane transport defect (THCMTD), thyroid hormone metabolism defect (THMD), and thyroid hormone action defect (THAD). THMD is caused by defects in the synthesis and processing of deiodinases that convert the prohormone thyroxine (T4) to the active hormone triiodothyronine (T3). Deiodinase, a selenoprotein, requires unique translation machinery that is collectively composed of the selenocysteine (Sec) insertion sequence (SECIS) elements, Sec-insertion sequence-binding protein 2 (SECISBP2), Sec-specific eukaryotic elongation factor (EEFSEC), and Sec-specific tRNA (TRU-TCA1-1), which leads to the recognition of the UGA codon as a Sec codon for translation into the growing polypeptide. In addition, THMD could be expanded to the defects of enzymes that are involved in thyroid hormone conjugation, such as glucuronidation and sulphation. Paucity of inherited disorders in this category leaves them beyond the scope of this review. This review attempts to specifically explore the genomic causes and effects that result in a significant deficiency of T3 hormones due to inadequate function of deiodinases. Moreover, along with *SECISBP2*, *TRU-TCA1-1*, and deiodinase type-1 (*DIO1*) mutations, this review describes the variants in *DIO2* single nucleotide polymorphism (SNP) and thyroid stimulating hormone receptor (*TSHR*) that result in the reduced activity of DIO2 and subsequent abnormal conversion of T3 from T4. Finally, this review provides additional insight into the general functionality of selenium supplementation and T3/T4 combination treatment in patients with hypothyroidism, suggesting the steps that need to be taken in the future.

## Introduction

Selenium as a basic element was first discovered by the Swedish chemist Jons Jacob Berzelius in 1817 (1, 2). Selenium is a trace element, and as such a “micronutrient” in humans and animals is generally obtained from the diet through food or other forms of supplementation (3, 4). We obtain dietary selenium in the form of selenomethionine (SeMet), selenocysteine (Sec), selenate, and selenite. Selenium metabolic systems play significant physiological roles in thyroid hormone metabolism, immunity, and antioxidant defense (4, 5). Selenium deficiency is associated with the occurrence, virulence, and progression of viral infectious diseases (6). In contrast, selenium supplementation resulted in immunostimulation, such as enhanced proliferation of activated T cells, activation of natural killer cells, and tumor regression mediated by cytotoxic lymphocytes (7, 8).

Selenium inadequacy is related to various types of diseases, such as cardiovascular disease (9–13), cancer (14–16), hepatopathy (17, 18), and arthropathy (19). Keshan disease is an endemic cardiomyopathy that occurs in selenium deficient areas in China and is prevented by sodium selenite supplementaion (9, 10, 12). Selenium deficiency is correlated with a significant increase in cancer incidence and mortality (14–16) and epidemiological evidence has accumulated on the cancer-preventing effects of selenium (20–22). Low selenium status is also characterized by liver injury (18) presumably resulting from elevated levels of oxidative stress (23). Oxidative stress caused by selenium deficiency plays a detrimental role in the development of joints (19). Selenium deficiency is the main cause of endemic Kashin–Beck disease (KBD), which is mainly presented as an arthropathy and reported in low-selenium areas of Far Eastern Asia. Furthermore, the pathogenesis of osteoarthritis (OA) may also be associated with oxidative stress caused by selenium deficiency (24–28).

Selenium is required for the production of thyroid hormone-metabolizing enzymes that are deiodinases, and selenium supplementation is thought to improve the function of thyrocytes (29). Impaired sensitivity to thyroid hormones, including genetic defects in thyroid hormone transport, metabolism, and action, describes disorders that interfere with the biological actions of thyroid hormone (30–32). Herein, we review the pathophysiology of impaired sensitivity to thyroid hormone. Of these, inherited disorders caused by thyroid hormone metabolism defects, mainly due to dysfunction of deiodinase, one of the selenoproteins, including the selenoprotein physiology, will be highlighted.

## Selenoprotein

### Selenium

**Figure 1:**
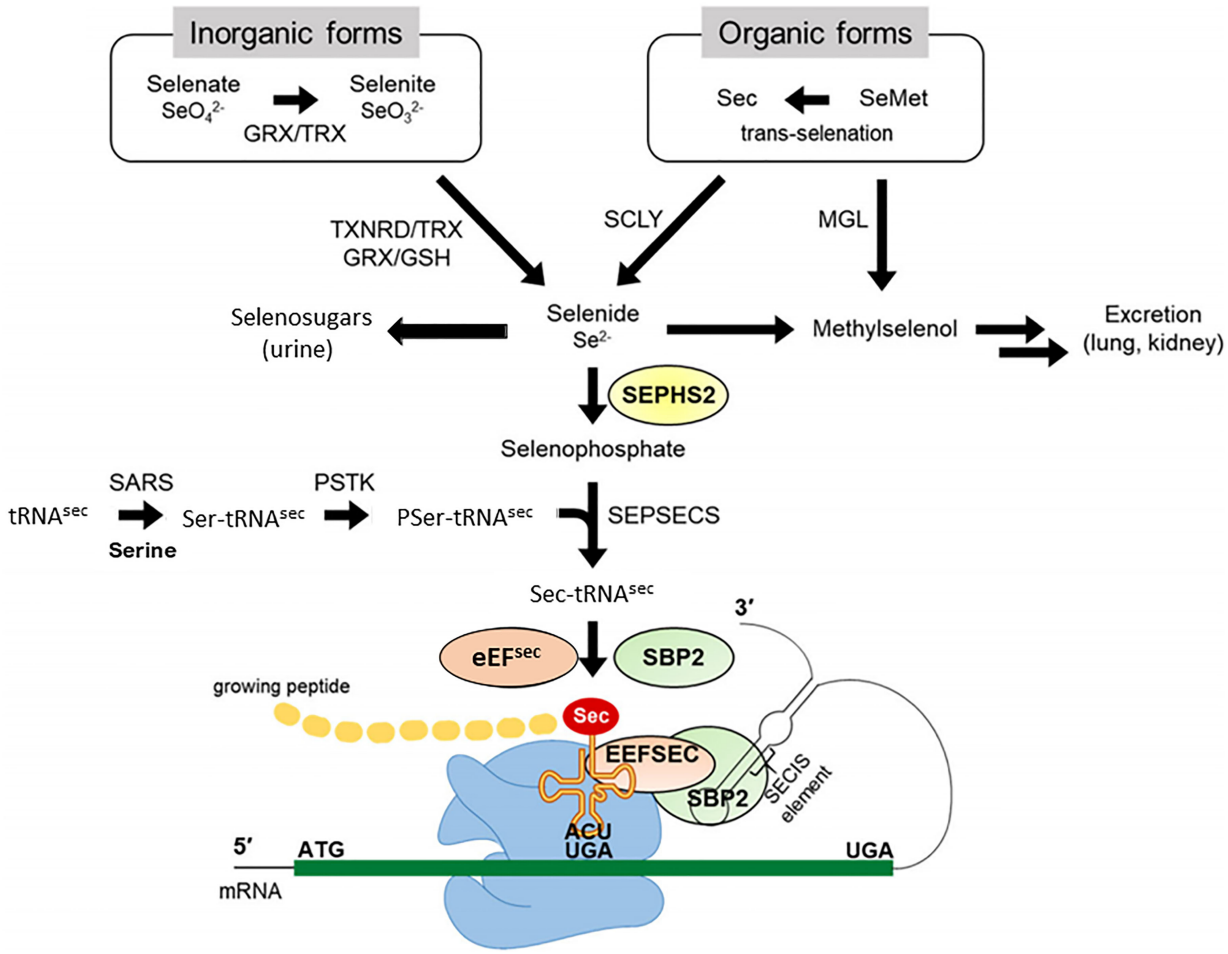
Selenium metabolic process in mammals. Selenium in organic forms, Sec and SeMet, and inorganic forms Selenate and Selenite taken from the diet, undergoes several conversion steps, and is incorporated into polypeptide chains, completing synthesis of selenoprotein. Selenide is synthesized from inorganic forms reduced by TXNRD/TRX or GRX/GSH systems and organic forms cleaved by SCLY. SEPHS2 synthesizes selenophosphate from selenide, and the subsequent reaction with PSer-TRU-TCA1-1 mediated by SEPSECS yields Sec-TRU-TCA1-1. SECISBP2 binds to SECIS located in the 3′UTR of a selenoprotein mRNA and mediates the transfer of Sec-TRU-TCA1-1 to the A-site of ribosome. Finally, Sec-TRU-TCA1-1 recognizes the UGA codon as the Sec integration codon. SeMet, selenomethionine; Sec, selenocysteine; GRX, glutathione reductase; TRX, thioredoxin; TXNRD, thioredoxin reductase; GSH, glutathione; MGL, methionine gamma-lyase; SCLY, selenocysteine lyase; SEPHS2, selenophosphate synthetase 2; SARS, seryl-tRNA synthetase; PSTK, phosphoseryl(Sep)-tRNA kinase; SEPSECS, Sep-tRNA: Sec-tRNA synthase; EEFSEC, Sec-specific eukaryotic elongation factor; SECISBP2, SECIS binding protein 2. Modified from Exp Mol Med. 2020 August; 52: 1198–1208.

Selenium is a trace element. Selenium metabolism systematically proceeds in the order of absorption, transportation, transformation, and excretion of selenium (**Figure 1**). Selenium is taken from the diet in organic forms, Sec and SeMet, and inorganic forms Selenate and Selenite. Selenium is absorbed by small intestine and taken up by the liver, which synthesizes and exports the selenoprotein P (SELENOP), ultimately circulating in the bloodstream. SELENOP, with a number of Sec residues, carries selenium to other tissues and organs, and the transported selenium is converted to selenophosphate *via* intracellular selenium metabolic pathway (33, 34). Selenium is excreted through exhalation and urine. Selenosugars are key urinary metabolites for selenium excretion within the required to low-toxic range (35–37).

Selenium plays a biological role mainly in the form of selenoproteins synthesized by the selenium metabolic system. Glutathione (GSH) and thioledoxin (TXN) systems first reduce ingested inorganic selenium to hydrogen selenide (H2Se). Sec amino acids converted from selenide are incorporated into specific sites of selenoproteins, such as the catalytic sites of the selenoenzyme. Mechanistically, selenophosphate is produced by the catalytic action of selenophosphate synthetase 2 (SEPHS2) through the reduction of hydrogen selenide. The subsequent reaction with phosphoseryl-tRNA (PSer-TRU-TCA1-1) yields Sec-TRU-TCA1-1. The intracellular machinery utilizing the UGA codon incorporates Sec amino acids into polypeptide chains. Selenocysteine insertion sequence binding protein 2 (SECISBP2) binds to the selenocysteine insertion sequence (SECIS) element located in the 3’-untranslated region (UTR) of selenoprotein mRNA, mediating the transfer of Sec-TRU-TCA1-1 to the A-site of the ribosome, which recognizes the UGA codon for integration of Sec. The selenoprotein translation machinery is collectively composed of SECIS elements, SECISBP2, Sec-specific eukaryotic elongation factor (EEFSEC), and aminoacylated Sec-TRU-TCA1-1, which can make the UGA codon recognized as a Sec codon and can be translated into the growing polypeptide (19).

### Selenocystein

Selenocystein (Sec) is the 21st proteinogenic amino acid, which was discovered by biochemist Thressa Stadtman at the National Institutes of Health (2, 38). Sec is a cysteine analogue with a selenium-containing selenol group in place of the sulfur-containing thiol group. After a long standing investigatory period, Sec has been confirmed as a new proteinogenic amino acid only after TRU-TCA1-1 was identified and characterized first in procaryote and later in mammalians (39–42).

### Selenoprotein

Selenoprotein is a protein containing a Sec amino acid residue. The biological function of selenium is mostly demonstrated through the selenoprotein domain, which contains Sec residues. Twenty-four selenoproteins have been identified and characterized in mice. Targeted deletion of these selenoproteins has revealed their essential roles in developmental processes and in the pathogenesis of diseases (43). In the human genome, 25 selenoprotein genes have been identified so far (44). Selenoproteins can be classified into subfamilies based on their cellular functions, such as anti-oxidation [Glutathione peroxidase (GPX)-1, GPX2, GPX3, and GPX4], calcium metabolism (SELENOK, SELENOT), myogenesis (SELENON), protein AMPylation (SELENOO), protein folding (SELENOF, SELENOI, SELENOS), redox regulation [thioredoxin reductase (TXNRD)-1, TXNRD2, TXNRD3, methionine sulfoxide reductase (MSRB)-1, SELENOH, SELENOM, SELENOW], selenium transport and storage (SELENOP), selenophosphate synthesis (SEPHS2), and thyroid hormone metabolism [deiodinase (DIO)-1, DIO2, DIO3] (45, 46). The cellular functions of other selenoproteins, such as GPX6 and SELENOV, remain to be elucidated. GPXs, such as GPX1 (cytosolic GPX), GPX2 (gastrointestinal GPX), and GPX4 (phospholipid hydroperoxide GPX), promote the decomposition of a wide variety of peroxides, protecting the cells from oxidative damage (47, 48). TXNRDs use NADPH as an electron donor to return oxidized TXN to a reduced dithiol, where oxidation states have a decisive effect on regulating various cell behaviors, including proliferation and apoptosis (49). The physiological importance of TXNRD is further supported by the embryonic lethality of either Txnrd1 or Txnrd2 knockout mice (50, 51). DIOs regulate thyroid hormone metabolism by catalyzing the conversion of thyroid hormones from precursor thyroxine (T4) to biologically active triiodothyronine (T3) or inactive reverse T3 (rT3) (52). The expression levels of some selenoproteins are affected by the degree of selenium intake. Selenium deficient animals and human cell lines, for example, have decreased the transcription of selenoproteins, such as GPX1, DIOs, SELENOI, and SELENOW (53–55). Some selenoproteins, such as GPX1 and SELENOW, are more sensitive to selenium supplementation or deficiency. The hierarchy of selenoprotein expression is more obvious at a limited intracellular selenium level (3). Selenium deficiency in cells in culture has also shown to reduce selenoprotein transcript levels by nonsense-mediated decay (56).

## Thyroid Hormone Physiology

**Figure 2:**
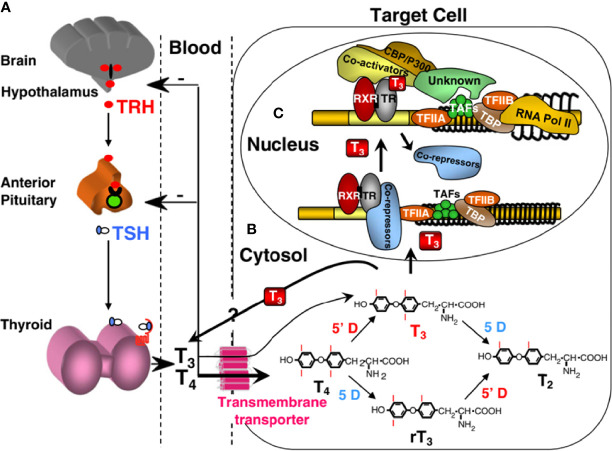
Regulation of TH supply, metabolism and genomic action. **(A)** Central feedback control that regulates the amount of TH in blood. **(B)** Intracellular metabolism of TH, regulating TH bioactivity. **(C)** Genomic action of TH. CBP/P300, cAMP-binding protein/general transcription adaptor; TFIIA and TFIIB, transcription intermediary factor II, A and B; TBP, TATA-binding protein; TAF, TBP-associated factor. Modified from Refetoff S, Dumitrescu AM. Syndromes of reduced sensitivity to thyroid hormone: genetic defects in hormone receptors, cell transporters and deiodination. Best Pract Res Clin Endocrinol Metab. 2007 Jun;21(2):277–305.

Homeostatic regulation of the thyroid hormone economy is tightly maintained by a feedback control mechanism involving the hypothalamus, pituitary, and thyroid gland (H-P-T) axis, as shown in **Figure 2**. As thyroid hormones are inhibitory for H-P-T axis, the decreased supply of thyroid hormones decreases the inhibitory effect on this axis leading to its increased activity. Conversely, excess supply of thyroid hormones shuts down the system through the same H-P-T axis pathway, resulting in a restored steady state. Fine tuning of the local thyroid hormone requirement is controlled through three additional steps. First, thyroid hormone entry across the cell membrane through transmembrane transporters such as MCT8 and MCT10 through facilitated diffusion (57–59). Second, the formation of active T3 (triiodothyronine) by removal of one of the outer ring iodine atoms (5’-deiodination) from prohormone T4 (thyroxine), or inactive rT3 and T2 by inner ring (5-deiodination) from T4 and T3, respectively, provide additional levels of control (52). Finally, the integrity of thyroid hormone receptors (THRs), through which thyroid hormone action is mediated, determines the type and extent of thyroid hormone response. Thyroid hormone action occurs not only in the nucleus of the target cell, but also in the cytoplasm (60, 61). The former, known as a genomic effect, has been studied extensively (62, 63). There are two THRs (THR-alpha and THR-beta) encoded by separate genes located on chromosomes 17 and 3, respectively. Different isoforms are formed by alternative transcription and splicing. The receptors have structural and sequence similarities with DNA binding and T3 binding domains. Other regions of the molecules are involved in dimeric formation with another THR or another type of nuclear receptor, and in binding coactivators and corepressor cofactors (64, 65). In the nucleus, THRs act as transcription factors that regulate the expression of certain genes, which are recognized through the thyroid hormone response element. Binding of unliganded dimeric receptors (without T3) to the thyroid hormone response element and recruitment of corepressor proteins results in inhibition of the expression of genes that are positively regulated by T3. When T3 binds to a receptor, the THR molecule undergoes a steric change, resulting in the release of corepressor proteins, dissociation of dimers, and formation of heterodimers of THR and retinoid X receptors that then bind coactivator proteins. This change promotes the expression of target genes and ultimately increases the synthesis of certain proteins. Although THRs reside primarily in the nucleus, they shuttle rapidly between the nucleus and cytoplasm. Recently, cryptic cytoplasmic functions have been described to other THR subtypes, expanding the diverse cellular responses to thyroid hormone. (66). Thyroid hormones can also act through binding sites at plasma membrane such as integrin αvβ3 (67).

## Syndromes of Impaired Sensitivity to Thyroid Hormone

Impaired sensitivity to thyroid hormone refers to any process that interferes with the effects of thyroid hormone, including defects in the transport, metabolism, or action of thyroid hormone (30, 68–70). Each defect is named representing the step affected as detailed in the following sections: (32, 71, 72).

### Thyroid Hormone Cell Membrane Transporter Defect (THCMTD)

Defects in one of the cell transport proteins that allow thyroid hormones to enter cells can cause decreased intracellular levels of thyroid hormones. Defective cell transport proteins may not locate their normal sites on the cell membrane or transport hormones. This causes a disorder depending on the hormone transporter that is affected. For example, a defect in monocarboxylate transporter 8 (MCT8) results in elevated serum concentrations of T3 and low levels of T4 and rT3 (68). This transporter is also involved in thyroid hormone secretion from the thyroid gland (73).

### Thyroid Hormone Metabolism Defect (THMD)

T4, a major form secreted by the thyroid gland, is a prohormone that must be converted to active T3 in the cytoplasm. Any defect in the factors involved in this enzymatic deiodination reaction can reduce T3 production, impairing the sensitivity to thyroid hormones. Abnormalities in the synthesis or degradation of various deiodinases may be included in these defects. Patients in this category had low serum T3 and high T4 and rT3 concentrations. More details in this category of defects will be described in section 5.

### Thyroid Hormone Action Defect (THAD)

Theoretically, for the thyroid hormone to enter the target cell to exhibit genomic effects, it must be transported into the nucleus and form a complex with the thyroid hormone receptor (THR) along with a series of cofactors to regulate the transcription of target genes. Therefore, it can interfere with the action of thyroid hormones due to disorders arising from defects in nuclear migration and various cofactors, but there have been no reports of patients showing such disorders in actual clinical practice.

Genomic thyroid hormone action is mediated through THRs, which act as transcription factors that activate or repress the transcription of certain target genes. Most RTHs are caused by THR defects (30). Mutant THR proteins have a reduced ability to bind cognate ligands or protein cofactors or bind to target genes. Mutations in the thyroid hormone receptor beta gene (*THRB*), which encodes the thyroid hormone receptor beta (THR-beta), are the most common cause of RTH and are defined as RTH-beta (74). In contrast, RTH-alpha, caused by mutations in the thyroid hormone receptor alpha gene (*THRA*), encoding thyroid hormone receptor alpha (THR-alpha), has rarely been reported (75).

## Inherited Defects of Thyroid Hormone Metabolism

### *SECISBP2* Mutations

Since SECISBP2 is epistatic to selenoprotein, SECISBP2 defects lead to low expression levels of selenoproteins due to poor Sec insertion and UGA decoding. To date, 12 families with *SECISBP2* mutations have been identified. Three of them had homozygous alleles, while nine had compound heterozygous alleles. Altogether, 20 unique *SECISBP2* mutations have been reported (70, 76–82)

Nearly all families from published cases exhibit common TFT abnormalities: elevated free T_4_ (FT_4_), elevated rT_3_, low free T_3_ (FT_3_), and normal or slightly elevated TSH levels. Such abnormalities are a universal indication of dysfunctional T_4_ to T_3_ conversion due to deficient DIOs from epistatic *SECISBP2* mutations (83).

Other biochemical signatures include low serum selenium levels, reflecting deficiencies of SELENOP and GPX3. Similarly, all but one patient (Family 5) exhibited short stature and delayed skeletal development (78). These are the most common clinical features of *SECISBP2* mutations. Growth retardation in *Dio2* and *Dio3* null mice suggests that the human phenotype is also mediated by abnormal thyroid hormone metabolism (84, 85). For more severe cases, failure to thrive was observed, while Family 4, arguably the most extreme case, exhibited multiple skeletal abnormalities such as craniofacial dysmorphism, bilateral clinodactyly, kyphoscoliosis, and leg asymmetry (77).

Symptoms unrelated to the skeleton and growth vary greatly depending on the severity of the mutations. Among the published cases, six suffered from delayed motor milestones and muscle weakness, especially in the legs (77–81). Families 5, 6, and 7 all exhibit characteristic phenotypes of *SEPN1* myopathies due to defects in SELENON, affected axial muscles, adductors, and sartorius (78, 79). Additionally, families 7 and 10 have connective or fatty tissue infiltrating the adductor muscles (79, 81). In some cases, myopathy is severe enough to exhibit Gowers’ signs or a waddling gait. All families suffering from myopathy, except for family 6, also suffer from neurological symptoms (78). Intellectual disability is the most common, while families 5 and 10 each have milder neurological symptoms: delayed motor milestones and attention deficit disorder, respectively (78, 81). Neurological phenotypes are difficult to associate with a deficit of any specific selenoprotein.

In severe cases, bilateral hearing loss (either sensorineural or conductive), secretory otitis media, and rotatory vertigo have been observed (77–79). One hypothesis associates this phenotype with a DIO2 deficit (84). However, a more recent alternate hypothesis suggests that the buildup of reactive oxygen species (ROS) due to antioxidant GPX1 deficiency leads to cochlear damage and hearing loss (78).

Common metabolic phenotypes such as elevated fat mass index, obesity, and paradoxically increased systemic sensitivity to insulin have also been observed in adults and children (78). *Gpx1* null mice exhibiting increased ROS levels become insulin sensitive (86). In contrast, mice overexpressing *Gpx1* develop insulin resistance (87). Thus, it is likely that these phenotypes are the result of increased cellular ROS due to GPX1 deficiency. Overall, a link between selenoproteins and systemic insulin sensitivity has been suggested. Family 10 was noted as the patient exhibited impaired glucose tolerance despite GPX1 deficiency (81). These conditions are assumed to be independent of *SECISBP2* mutations.

The only adult in the studies, proband of family 5, exhibits unique phenotypes such as azoospermia and photosensitivity (78). Azoospermia is likely a result of the underexpression of GPX4, thioredoxin/glutathione reductase (TGR), and selenium-containing protein V (SELENOV), the first two of which are integral in sperm development. Cutaneous photosensitivity is attributed to the underexpression of the antioxidant selenoprotein, which increases susceptibility to ROS generation from UV rays. The other characteristics and clinical features are summarized in **Table 1**.

**Table 1:** Selenoprotein mutations affecting thyroid hormone metabolism defects (THMDs) and their clinical features.

Family # (Reference)	Mutations	Protein change	Thyroid hormone metabolism				Skeletal structure and growth	Muscular and neurological effects	Hearing and balance	Metabolic effects and others	Status
			FT_4_	rT_3_	FT_3_	TSH					
***SECISBP2***											
1 (70)	c.1619G>A	p.R540Q	↑	↑	↓	↑	Short stature, delayed bone age	–	Normal	–	Homozygous
2 (70)	c.1312A>T	p.K438X	↑	↑	↓	normal	Short stature, transient growth retardation	–	Normal	–	Compound heterozygous
	c.1283+29G>A, abnormal splicing	Frameshift									
3 (76)	c.382C>T	p.R128X	↑	↑	↓	normal	Short stature, delayed bone age	–	–	–	Homozygous
4 (77)	c.358C>T	p.120X	↑	↑	↓	↑	Short stature, delayed bone age. Failure to thrive. Craniofacial dysmorphism. Bilateral clinodactyly, short fifth metacarpals. Kyphoscoliosis, leg asymmetry	Hypotonia, hyporeflexia, limited flexion of the neck. Symmetrical generalized peripheral sensitive neuropathy in the legs. Hip girdle weakness, waddling gait, Gower’s sign. Impaired motor coordination. Intellectual disability	Bilateral sensorineural hearing loss	Obesity. Protruding tongue. Left eye semiptosis	Compound heterozygous
	c.2308C>T	p.R770X									
5 (78)	c.668delT	p.F223F fs X32	↑	↑	normal	normal	Genu valgus, external rotation of the hip	Muscle weakness, reduced aerobic exercise capacity, reduced lung vital capacity. Abnormal spinal curvature, fatty infiltration. Delayed motor and speech developmental milestones	Bilateral sensorineural hearing loss. Secretory otitis media. Rotatory vertigo	Fatigue. Severe Raynaud disease. Azoospermia. Photosensitivity. Persistent reduction in rbc and total lymphocyte counts. Elevated fat mass index, increased insulin sensitivity	Compound heterozygous
	c.881-155T>A, abnormal splicing	Frameshift									
6 (78)	c.2071T>C	p.C691R	↑	↑	↓	normal	Short stature, delayed development. Failure to thrive	Muscle weakness, hypotonia, lumbar spinal rigidity, nasal voice. Delayed motor milestones	Bilateral sensorineural hearing loss	Nonketotic hypoglycemia. Eosinophilic colitis. Elevated fat mass index, increased insulin sensitivity	Compound heterozygous
	Intronic SNP, abnormal splicing	Frameshift									
7 (79)	c.1529_1541dupCCAGCGCCCACT	p.M515Q fs X48	↑	–	↓	normal	Short stature, delayed development. Failure to thrive	Delayed motor and intellectual milestones. Fatty infiltration. Intellectual disability	Bilateral conductive hearing loss. Secretory otitis media. Rotatory vertigo	Fatigue. Bilateral hyperopia, esotropia. Hypoplastic thyroid gland	Compound heterozygous
	c.235C>T	p.Q79X									
8 (80)	c.2344C>T	p.Q782X	↑	↑	↓	normal	Delayed development	Delayed motor and intellectual milestones. Intellectual disability’	Normal	–	Compound heterozygous
	c.2045-2048 delAACA	p.K682 fs 683X									
9 (82)	c.589C>T	p.R197X	↑	↑	normal	normal	Short stature, delayed development. Failure to thrive	–	–	–	Compound heterozygous
	c.2037G>T	p.E679D									
10 (81)	c.2045-2048	p.K267K fs X2	↑	↑	↓	normal	–	Leg weakness, Gowers’ sign. Fatty infiltration. Attention deficit disorder	–	Obesity, impaired glucose tolerance. Fatigue. Right eye ptosis	Homozygous
11 (82)	c.1588A>G	p.T530A	–	–	–	–	–	–	–	–	Compound heterozygous
	c.1711C>T	p.Q571X									
12 (82)	c.283delT	p.Y95I fs X31	–	–	–	–	–	–	–	–	Compound heterozygous
	c.589C>T	p.R197X									
***TRU-TCA1-1***											
13 (88)	C65G		↑	↑	normal	normal	–	Muscle weakness	–	Fatigue. Abdominal pain.	Homozygous
***DIO2* and *TSHR***											
14 (89)	*DIO2* c.274A>G	p.T92A	↓	–	↓	↑	–	–	–	Anorexia and weight gain. Hypothyroidism	Homozygous*
	*TSHR* c.1349G>A	p.R450H									Heterozygous*
15 (89)	*DIO2* c.274A>G	p.T92A	↓	–	↓	↑	–	–	–	Thyroid goiter, congenital hypothyroidism	Homozygous
	TSHR c.1574T>C	p.F525S									Heterozygous
***DIO1***											
16 (90)	c.282C>A	p.N94K	–	↑	↓**	↑	–	–	–	Down syndrome	Heterozygous
17 (90)	c.603G>A	p.M201I	–	↑	↓**	–	–	–	–	Resistance to TRH. Elevated cholesterol	Heterozygous

*The proband has a homozygous deiodinase type-2 (DIO2) mutation and a heterozygous thyroid-stimulating hormone receptor (TSHR) mutation, whereas his affected grandson has a heterozygous DIO2 mutation and a homozygous TSHR mutation.

**Free T_3_ (FT_3_) levels by themselves were never specified. The probands were tested for reverse T_3_ (rT_3_)/FT_3_ ratio.

### *TRU-TCA1-1* Mutation

Homozygosity mapping with known consanguinity in the proband (an 8-year-old boy who exhibited thyroid dysfunction [raised T4, normal T3, raised reverse T3]) identified a single interval in the proband, encompassing the chromosomal locus of only one gene (*TRU-TCA1-1*) in the Sec-incorporation pathway. Sequencing of this gene in the proband indicated homozygosity for a single nucleotide change, C65G in *TRU-TCA1-1* (88). The early stages of tRNA^Sec^ maturation involve sequential base modifications, yielding two major tRNA^Sec^ isoforms containing either 5-methoxycarbonyl-methyluridine (mcm^5^U) and 5-methoxycarbonyl methyl-2’-O-methyluridine (mcm^5^Um) at position 34, situated in the anticodon loop. Isoform containing mcm^5^U is known for its role in synthesizing housekeeping selenoproteins, while another isoform containing mcm^5^Um is known to be responsible for the synthesis of stress-related selenoproteins (91, 92). Primary cells from the proband showed a marked reduction in the mcm^5^Um isoform of tRNA^Sec^, whereas the mcm^5^U isoform of tRNA^Sec^ was relatively preserved. Radiolabeled tRNA^Sec^ injection into *Xenopus* oocytes followed by chromatography analysis indicated significantly attenuated base modification in C65G *TRU-TCA1-1* compared with the wild-type, indicating weakened subsequent methylation of mcm^5^U at position U34. This confirms that the maturation of mutant tRNA^Sec^ is impaired. These findings indicate that a *TRU-TCA1-1* mutation can selectively impair the synthesis of stress-related selenoproteins (88).

### *DIO2* SNP and *TSHR* Mutations

**Figure 3:**
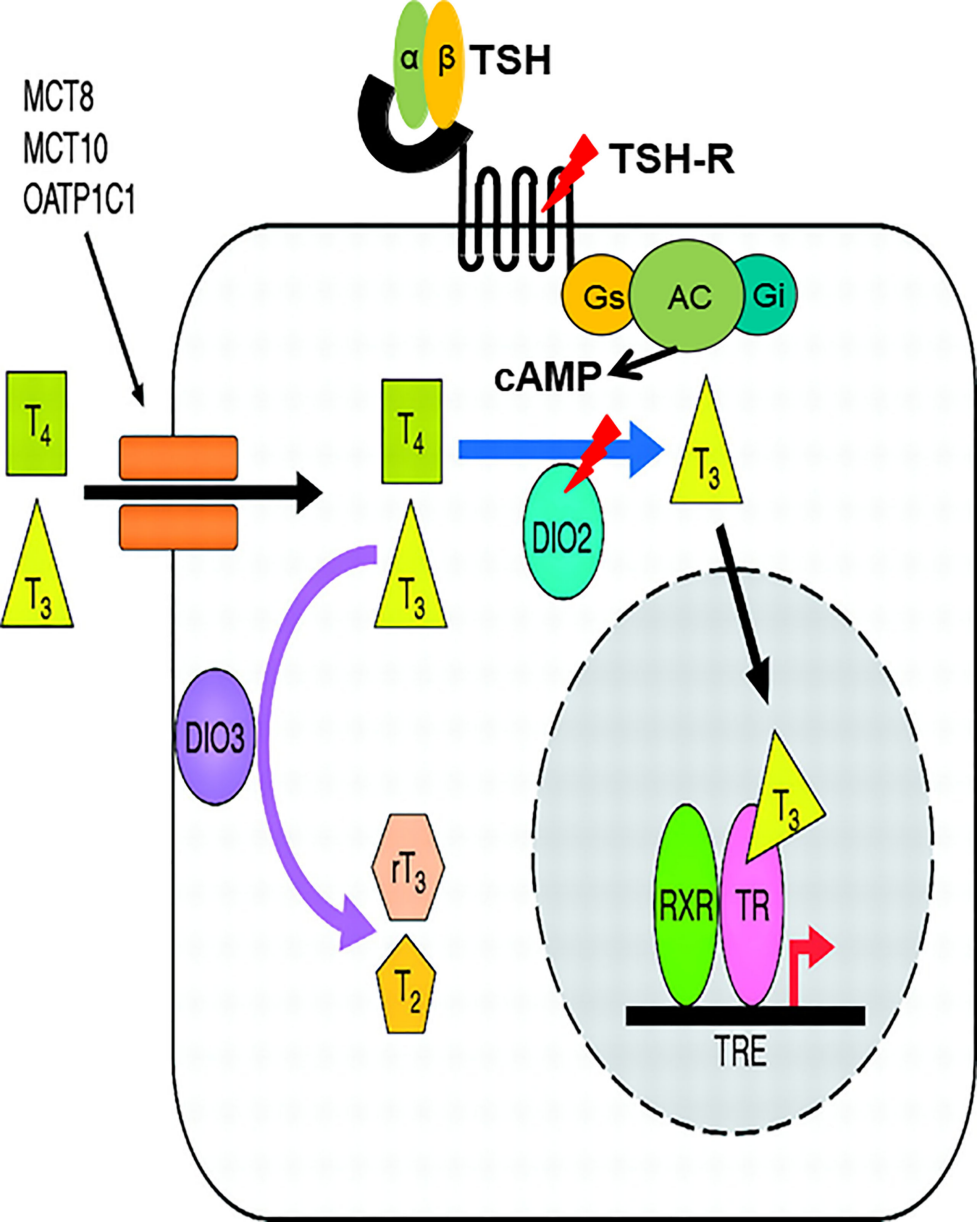
TSHR loss-of function mutations and homozygous DIO2 T92A SNP (“double hit”) cause decreased activity of DIO2, resulting in a novel form of abnormal thyroid hormone metabolism. cAMP produced by interaction of TSH and TSHR affects the cAMP response element located upstream of promoter region of DIO2. Lack of cAMP production caused by loss-of-function mutation of TSHR and DIO2 T92A SNP cooperatively causes decreased DIO2 enzymatic activity.

In an extensive case-finding study conducted in Korea, which targets patients who present inappropriately high TSH levels despite a high dose of levothyroxine (L-T4) and a drop in TSH levels only after addition of liothyronine (L-T3), two patients were noted (89). The first is a 68-year-old Asian man with symptoms of anorexia and weight gain for three months. Initial serum TSH was 85.50 mIU/L (0.27–4.20) and FT_4_ was 0.16 ng/dL (0.93–1.70). After diagnosis of hypothyroidism, he began to take L-T4 at 100 μg per day, but his serum TSH level was not suppressed. When L-T3 30 μg was administered three times a day instead of L-T4 100μg, the serum TSH level dramatically decreased. The second patient was a 75-year-old Asian woman who had suffered from a huge thyroid goiter (up to 10 cm in diameter) since her early twenties. Her thyroid function tests revealed extremely high TSH levels, inappropriately high FT_4_ and relatively low T3 levels; TSH 82 mIU/L, FT_4_>7.70 ng/dL, T3 64 ng/dL without thyrotoxic symptoms. Her goiter decreased in size dramatically when administered L-T3 30 μg twice a day, followed by a decrease in TSH level to 8.21 mIU/L and an increase in T3 level to 94 ng/dL without differences in FT_4_ levels. The TRH stimulation test revealed that it was less likely due to either RTH-or TSH-secreting pituitary adenoma for inappropriately increased TSH. Nucleotide sequencing of the first patient revealed a homozygous SNP in the *DIO2* gene (c.274A>G, DIO2 T92A), which had previously been identified in a certain portion of the population as a significant SNP (93) and a heterozygous mutation in the *TSHR* gene (c.1349G>A, R450H) (94). Interestingly, the second patient also had a homozygous DIO2 T92A mutation, together with a heterozygous TSHR F525S mutation. Both TSHR R450H and F525S are loss-of-function mutations. Since it was first reported that DIO2 is regulated by TSH (95, 96), there have been reports supporting the same concept in various cell types (97, 98). Large cohort studies with normal thyroid function also support the notion that TSH regulates DIO2 both in pediatric and adult populations (99, 100). In the same context, one can hypothesize that decreased signal from TSH due to defects in TSHR as well as DIO2 dysfunctional SNP results in reduced activity of DIO2 and abnormal conversion of T3 from T4 (**Figure 3**). To prove this hypothesis, DIO2 activity was measured using fibroblasts obtained and primarily cultured from patients harboring the DIO2 T92A SNP together with the TSHR R450H mutation. Both the relative activity and mRNA expression of DIO2 were blunt in fibroblasts from the patient, confirming the hypothesis (89).

### *DIO1* Mutations

Next generation sequencing analyses of two patients from two unrelated families presenting with abnormal TH metabolism and elevated serum rT3 levels and rT3/T3 ratios revealed two missense DIO1 mutations (p.Asn94Lys and p.Met201Ile) (90). *In silico* prediction of these DIO1 mutants suggested pathogenic variants, and *in vitro* assessment of the functional activity of DIO1 mutants showed decreased enzymatic activities, confirming that this mutation is disease-causing. Heterozygous *Dio1* knock-out mice back crossed >10 times into the WT 57BL/J5 strain showed elevated serum rT3 levels and elevated rT3/T3 ratios, consistent with the data observed in the affected patients. Advances and widespread sequencing technologies will allow us to identify more mutations in patients showing a bizarre thyroid function test.

## Selenium Supplementation

### Healthy Population

The effect of selenium deficiency and selenium supplementation on general health, especially on thyroid function, remains to be elucidated (29). Several trials have been conducted in regions with different baseline selenium status in UK, New Zealand, USA, and rural Tibet (101–105). No definite effects or consistent adverse effects on thyroid function have been observed (102). In critical illnesses, such as severe sepsis or trauma injuries, selenium supplementation showed null or only moderate effects on thyroid function (106, 107). These findings suggest that selenium supplementation does not play an important role in determining the serum thyroid hormone status. Meanwhile, selenosis, i.e., an excess of Se intake, should be avoided, as Se in high dosages is toxic, potentially due to elevated selenoprotein P levels as observed in clinical trials with therapeutic dosages of selenite.

### *SECISBP2* Mutations

Identification of the clinical phenotypes of affected patients and the metabolic pathways responsible for them provides insight into targeted treatment options. Selenium supplementation could first be considered an ideal treatment modality for disorders caused by selenium and selenoprotein dysregulation. Administration of up to 400 µg of selenomethionine-rich yeast, but not sodium selenite, normalized the serum selenium concentration but did not recover normal thyroid hormone metabolism in patients with *SECISBP2* mutations (108). The difference in Selenium bioavailability between selenomethionine and selenite results from the efficiency of Selenium incorporation. Selenomethionine seems to be more efficient because it can be non-specifically incorporated into all circulating serum proteins (109), whereas selenite is metabolized and inserted as Sec into the growing peptide chain of selenoproteins (110).

### Autoimmune Thyroid Diseases

Hashimoto’s thyroiditis and Graves’ disease are the most common autoimmune thyroid diseases (111). Reduced serum selenium concentrations have been reported in patients with autoimmune thyroid disease (112, 113). Consequently, several studies have tested the efficacy of selenium supplementation in patients with Hashimoto’s thyroiditis. Three trials reported successful reductions in TPO autoantibodies titers and/or improvement of mood and well-being (114–117). As no consistent adverse events were observed, routine selenium supplementation in patients with Hashimoto’s thyroiditis was considered as a promising adjuvant treatment option (118). It is worth noting that experiences with subjects deficient in both iodine and selenium, and displaying increased disease symptoms when Se supplementation was initiated without at the same time raising iodine supply (so called myxedematous cretinism).

Selenium supplementation trials in patients with Graves’ disease are limited. One of the major and severe complications of Graves’ disease, ophthalmopathy (also known as orbitopathy) is an inflammatory process in nature and presents as a protrusion of one or both eyes. Increased intraorbital pressure results in proptosis and compressive neuropathy, and inflammation of the extraocular muscles causes diplopia (119). One randomized clinical trial of selenium supplementation in patients with mild Graves’ orbitopathy was performed, and the quality of life and eye disease parameters improved significantly after 6 months of treatment (120, 121). Recently, it was reported that selenium suppressed hyaluronan production, inflammatory cytokines, and intracellular ROS generation in cultured orbital fibroblasts of patients with Graves’ orbitopathy (GO), suggesting a basis for the use of selenium in the treatment of GO (122).

## Thyroid Hormone Replacement

Approximately 5–10% or more of biochemically well-controlled hypothyroidism patients with levothyroxine (L-T4) treatment have persistent complaints, such as depression and impaired psychological wellbeing (123). There are various explanations for the discrepancies between thyroid function tests and clinical symptoms, but there is no definite answer yet. Instead, expectations for T3/T4 combination therapy have been raised as a realistic solution. Several trials using combined T3/T4 therapy have been conducted for comparison with L-T4 monotherapy in the past years. Some studies have shown a beneficial effect, such as patient preference or an improved metabolic profile (124–127), however, in general, patients on T3/T4 combination therapy do not have improved outcomes compared with those on L-T4 monotherapy (128, 129). Possible explanations for unsatisfactory results may include inadequate L-T4 and L-T3 doses or frequency of administration (130).

Moreover, individuals with genetic variations in thyroid hormone metabolism should be considered (130). A subgroup that could be targeted is individuals with common genetic variations in DIO2, such as T92A SNP, which encodes the deiodinase 2 enzyme that converts T4 to T3 locally in several tissues (131). In a study conducted in the UK, the T92A SNP in DIO2 was associated with lower baseline psychological well-being in patients on LT4 replacement therapy and with better response to T3/T4 combination therapy, compared with patients without the SNP on L-T4 replacement therapy (132). In contrast, results from a large population-based cohort study revealed no effect of the T92A SNP on quality of life or cognitive function measures (133).

Generally, L-T_3_ is much more effective in treating developmental symptoms in patients with SECISBP2 mutations. For example, delayed linear growth can be improved by L-T3 supplementation (76). Similarly, L-T_3_ improved growth, speech, and development, while normalizing TFT (78). Of particular interest is the treatment of Japanese patients (79). The proband was initially treated with GH alone, since 52 months of age, in the hope of normalizing the TFT and improving his growth. While six years of GH treatment increased his height SDS from -3.4 to -1.7, his TFT abnormalities persisted. Addition of L-T_3_ to the treatment for six months normalized TFT and advanced bone age. Later treatment of the proband with α-tocopherol (vitamin E) was shown to have decreased lipid peroxidation products and increased circulating white blood cells and neutrophils, strengthening the immune system.

The effect of L-T3 administration was also tested in two patients, as it was demonstrated to equally suppress serum TSH concentration, which was not sufficiently suppressed by L-T4 in T92A SNP in DIO2 and TSHR mutations (89).

While the American Thyroid Association guidelines generally disadvocate the routine use of T3/T4 combination therapy in patients with hypothyroidism (134), the European Thyroid Association guidelines state that a 3-month trial of T3/T4 combination might be considered experimentally in adherent, biochemically well-controlled patients who have persistent symptoms despite L-T4 treatment (135). Sufficiently powered prospective randomized controlled trials are therefore prerequisite before concrete conclusions can be drawn, especially considering the genetic variations responsible for thyroid hormone metabolism discussed in this review. Collectively, these findings could provide clinical relevance in a select population of hypothyroidism patients who might benefit from T3/T4 combination therapy.

## Conclusions and Future Directions

The main reason for the increased interest in rare genetic diseases and their molecular genetic mechanisms is to facilitate the effective treatment of more common diseases related to it. In the treatment of hypothyroidism, there has always been much controversy about T4 treatment and T3/T4 combination therapy. It is the Se status and Se intake that may be critical for success or failure of the T3/T4 combination therapy, in view of Se deficiency potentially impairing sufficient DIO expression in target tissues for efficient T3 production (without this defect being reflected in circulating thyroid hormone concentrations). It is expected that a fundamental approach to this will become possible as knowledge accumulates through future studies. As our understanding of selenoprotein metabolism and action deepens, the scope for its clinical application expands. In the future, it is expected that a new era of the most appropriate thyroid hormone replacement therapy will further expand our understanding of the molecular mechanisms of action of selenium and selenoprotein, especially the entire process of thyroid hormone metabolism through DIOs and its related pathological conditions.

## Author Contributions

KL and SiL contributed to conception and design of the study. KL, YS, and SuL collected references. KL wrote the first draft of the manuscript. KL, YS, and SuL wrote sections of the manuscript. SiL wrote the final version of the manuscript. All authors contributed to manuscript revision, read, and approved the submitted version.

## Funding

This study was supported by the Basic Science Research Program, National Research Foundation of Korea (grant number NRF-2019R1F1A1063459 to SiL) and Korea Research-driven Hospitals Grant fostered for Gachon University Gil Medical Center (grant number 2018-5284 to SiL).

## Conflict of Interest

The authors declare that the research was conducted in the absence of any commercial or financial relationships that could be construed as a potential conflict of interest.

## Publisher’s Note

All claims expressed in this article are solely those of the authors and do not necessarily represent those of their affiliated organizations, or those of the publisher, the editors and the reviewers. Any product that may be evaluated in this article, or claim that may be made by its manufacturer, is not guaranteed or endorsed by the publisher.
